# Understanding of the Effect of the Adsorption of Atom and Cluster Silver on Chitosan: An In Silico Analysis

**DOI:** 10.3390/molecules28155809

**Published:** 2023-08-01

**Authors:** Alejandro Rodríguez-Juárez, Veronica Carmona-Álvarez, Fernando Díaz-Monge, Ernesto Chigo-Anota, Orlando Zaca-Moran

**Affiliations:** 1 Tecnológico Nacional de México, ITS-Tlaxco, Predio Cristo Rey Ex-Hacienda de Xalostoc Carretera Apizaco-Tlaxco Km. 16.8, Centro, Tlaxco 90250, Mexico; vero94alva@gmail.com (V.C.-Á.); fernando.dm@tlaxco.tecnm.mx (F.D.-M.); 2 Facultad de Ingeniería Química, Benemérita Universidad Autónoma de Puebla, Ciudad Universitaria, San Manuel, Puebla 72570, Mexico; 3 Instituto Politécnico Nacional, Centro de Investigación en Biotecnología Aplicada, Ex-Hacienda de San Juan Molino, Km 1.5 de la Carretera Estatal Santa Inés Tecuexcomac-Tepetitla, Tepetitla 90700, Mexico; ozacam@ipn.mx

**Keywords:** chitosan, silver, DFT theory, electronic properties

## Abstract

In this work, the structural, electronic, and optical stability properties of the chitosan monomer (M-Ch) and atomic silver complex are reported, as well as a unitary cell of a silver cluster in the gas phase and acetic acid. The generalized gradient approximation HSEh1PBE/def2-TZVPP50 results established the structures’ anionic charge (Q = −1|e|) and the doublet state (M = 2). The high cohesive energy indicates structural stability, and the quantum-mechanical descriptors show a high polarity and low chemical reactivity. Also, the quantum-mechanical descriptors present a low work function that shows the structures are suitable for applications in light-emitting diodes. Finally, the electronic behavior observed by the |HOMO-LUMO| gap energy changes depending on the atomic silver incorporated into the complex.

## 1. Introduction

Presently, many polymers, among them biopolymers, have relevance within medicine, biochemistry, genetics, and materials engineering, due to their applications as fibers, adhesives, etc. These biopolymers are classified into polysaccharides (starch, chitin, chitosan, cellulose, and its derivatives), proteins (amino acids, enzymes, and peptides), and polynucleotides (polyesters of phosphoric acid and nucleotides). Because of this, the number of studies about biopolymers has increased to find or improve applications [1].

The scientific community’s attention is focused on finding natural resources to improve human health and wellness by investigating the reuse of seafood waste, e.g., the exoskeletons of crustaceans and fish scales. This seafood waste is considered an environmental pollutant. Nevertheless, it is the primary source of two worldwide value-added biopolymers, chitin and chitosan [2], with chitosan the most abundant on Earth owing to its extraction via the deacetylation of the chitin process, and comprising the main constituent of fishing waste [3,4].

Chitosan (Ch) is a natural polysaccharide composed of β-(1-4) glucosamine and N-acetyl glucosamine [5]. It has excellent properties, such as a chelating agent, easy-to-form fiber, good biocompatibility, biodegradability, non-toxicity, antibacteriality, and good anti-hemostatic activity [6,7,8]. In the pharmaceutical industry, it is used as a drug-delivery agent [9], and in the electronic industry, it is used as a microfluidic bio-chip, based on the chitosan-titania composite [10].

Metallic structures (Cu, Au, Pt, Cd, and Ag) and metallic structures combined with polymers have been employed to design and develop bionanosensors [11,12,13] to prepare medical, catalytic, and optical equipment [14,15]. The most studied are silver (Ag) nanoparticles, which are used as stabilizers, composites, biosensors, and antimicrobial agents, and for drug delivery [16,17,18], because of their potential absorption behavior [19]; Ag nanoparticles have even been used for cancer treatment. However, healthy cells undergo unfavorable effects when nanoparticles are below 20 nm [19,20]. Silver nanoparticles also present electronic and conductivity properties [21,22], but there needs to be more research into the electronic properties of silver nanoparticles and their interaction with chitosan (Ag–Ch). Therefore, this work analyzes the effect of the silver atom adsorption and a silver cluster on the chitosan monomer using Density Functional Theory to find potential applications.

## 2. Computational Aspects

The free software ArgusLab 4.0.1 was used to build geometrical atomic positions. Subsequently, Density Functional Theory (DFT) [23] was used to study the interaction between silver atoms (Ag), silver clusters (denoted by c and with a unitary cell composed of 14 atoms), and the chitosan monomer (MCh). For the analysis of the chitosan monomer, the HSEh1PBE functional [24,25] and the set 6-311G(d,p) basis were used (see Table A1). At the same time, the optimization process of the complex formed by the Ag and the MCh was carried out using two approaches. The first consisted of looking for the most stable configuration given the minimum energy criterion, where seven possible interaction configurations were analyzed considering the main functional groups of the monomer; this was carried out at the level of generalized gradient approximation with the hybrid functional HSEh1PBE and a DGDZVP basis set [26,27], as implemented in the GAUSSIAN 09 quantum chemistry software version 9.0 [28]. 

**Figure 1:**
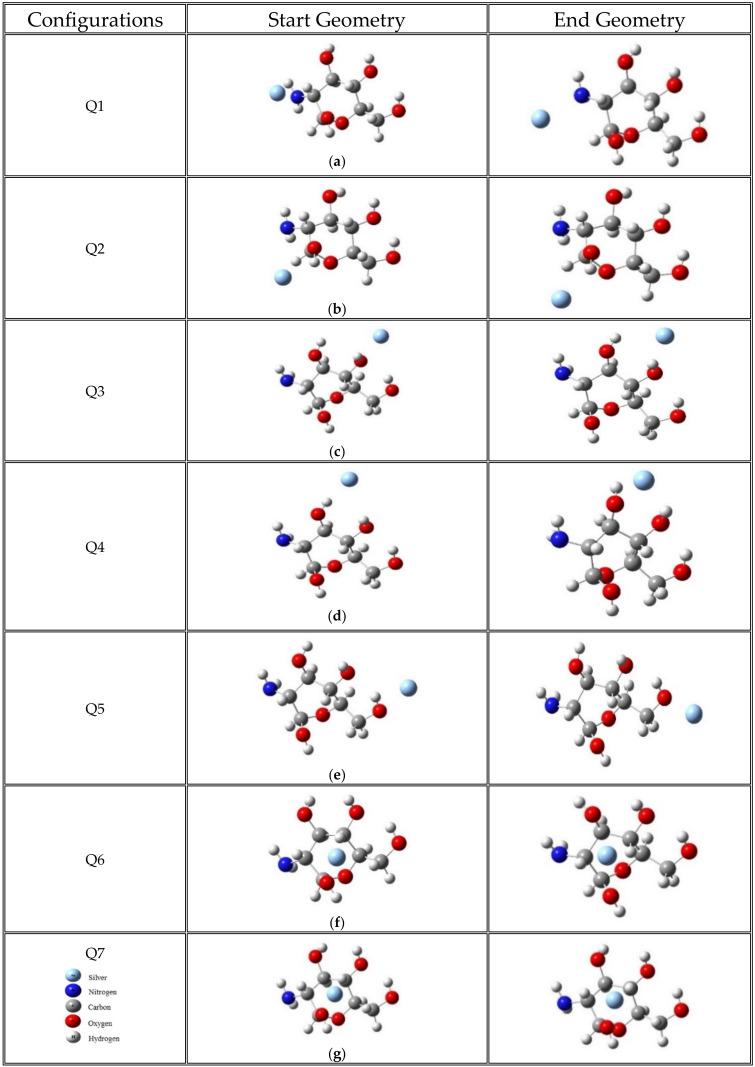
Initial and final geometries considering the interaction between the chitosan monomer and silver atom. (**a**) The silver atom with amino group interaction; (**b**,**c**,**e**) The silver atom with C–OH group interaction; (**d**) The silver atom with CH_2_OH group interaction; (**f**) The silver atom with oxygen from hexagonal ring interaction; (**g**) The silver atom is oriented at the center of the ring interacting with the OH group attached to the CH group (between the amino group and the O belonging to the ring). The initial distance in all cases was 2.0 Å.

The charge and multiplicity (M = 2S_T_ + 1, where S_T_ is the total spin) for chitosan were neutral and 1 (singlet state), respectively. By contrast, for the MCh–Ag complex, a neutral charge and multiplicity of 2 were considered. For the first structure (Q1) analyzed, silver is oriented towards the amino group of the monomer (N: blue sphere, C: gray sphere, O: red sphere, and H: white sphere, Figure 1a); in the second structure (Q2), the silver atom was positioned in the OH–CH functional group attached to the oxygen atom of the hexagonal ring (Figure 1b). In the third (Q3) and fourth (Q4) structures, the Ag atom is positioned on the opposite side of Q2 and onto the OH–CH group on the hexagonal ring (Figure 1c,d). The fifth structure (Q5) indicates that the Ag atom is positioned on the CH_2_–OH functional group of the MCh (Figure 1e). Finally, in the Q6 and Q7 systems (Figure 1f,g), the silver atom is positioned above and below the chitosan monomer hexagonal ring. In a second process, the effect of the charge (anion and cation) and the multiplicity (M = 2) for each system were analyzed in order not to find degenerate states concerning the energy of each system (Table A2). Also, the effect of a cluster of Ag atoms as an FCC unit cell interacting with MCh was studied. It is worth mentioning that the silver cluster was chosen considering an embryo in the crystalline phase, emulating a natural system situation at the nanometric level. However, there are reports in the literature where different clusters are studied [29,30,31], and the crystalline case has yet to be reviewed at this level of theory.

These structures were also re-optimized at the HSEh1PBE theory level, derived from the PBE method, and include 25% of the exact Hartree–Fock exchange. It can describe non-covalent interactions and the more extensive def2-TZVPP basis set [32] because this basis set describes organometallic with 4d and 5d elements very well. Also, in this way, to guarantee the determination of local minima, vibrational calculations within the harmonic approximation were carried out for the lowest energy structures, obtaining positive values. The systems presenting the lowest total energies are shown in Table A2.

A more detailed study has analyzed the quantum descriptors within the DFT framework [33]. The electronic energy gap (ΔΕ_gap_) was considered to be the energetic difference of the frontier orbitals—LUMO (lowest unoccupied molecular orbital) and HOMO (highest occupied molecular orbital)—as follows: ΔΕ_gap_ = (ε_LUMO_ − ε_HOMO_)/2 ≈ |ε_LUMO_ − ε_HOMO_|. The chemical potential, μ, a measure of global chemical reactivity, was estimated as the μ = (ε_HOMO_ + ε_LUMO_)/2) arithmetic mean [34]. For the design of optoelectronic devices, essential to obtain the work function (WF) [35], this parameter is defined as the minimum energy needed to remove an electron from a solid to a point immediately outside the solid surface or needed to move an electron from the Fermi energy level into the vacuum. The work function (WF) was estimated as the difference between the potential energy of the empty LUMO level and the Fermi energy (or chemical potential). The cohesion energy of these structures was determined as follows: E_Coh_ = E_T_(C_6_H_13_NO_5_Ag_14_) − kE_T_(Carbon) − pE_T_(Nitrogen) − nE_T_(Hydrogen) − gE_T_(Silver)/(k + p + n + g), where E_T_ represents the total energy for the most stable structure of a system (for Q = 0 and multiplicity = 1). E_T_ (carbon, nitrogen, hydrogen, and silver) represents the total energy for the ground state of the carbon, nitrogen, hydrogen, and silver atoms. The labels k, p, and g represent the amount of carbon, nitrogen, hydrogen, and silver atoms in the structures [36]. The MEP (Molecular Electrostatics Potentials) surfaces were determined as described in the literature [37]. These surfaces are usually associated with lone pairs of the more electronegative atom.

To obtain more realistic results, the systems were investigated in the presence of acetic acid simulated with the Polarizable Continuum Model (PCM) using one acetic acid dielectric constant (ε = 6.2528), as implemented in software GAUSSIAN 09 [28]

## 3. Results and Discussion

**Figure 2:**
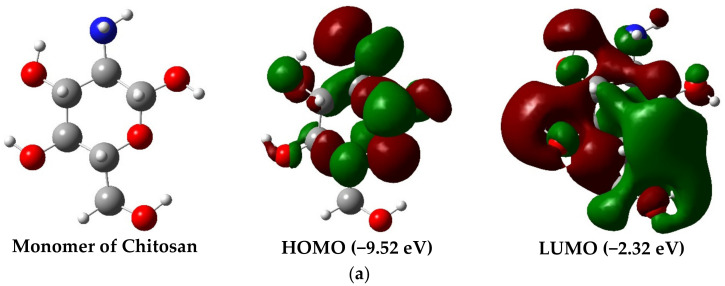

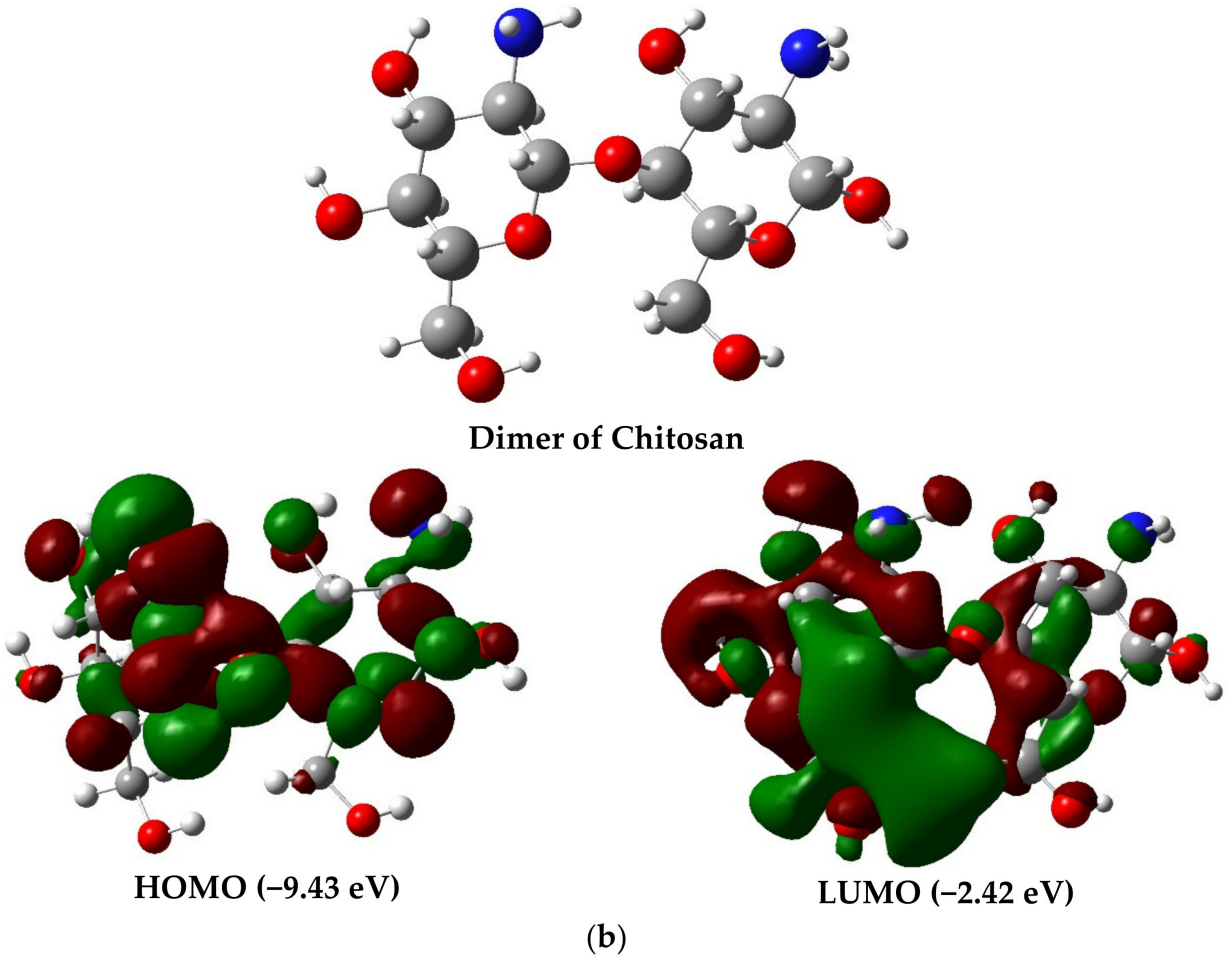
Optimized structures of (**a**) Chitosan monomer and (**b**) Chitosan dimer with HOMO and LUMO iso-surfaces, respectively.

The results of chitosan and the complex formed by chitosan and silver were analyzed as follows: first, it was investigated whether a significant effect to consider in the chitosan monomer or dimer (Figure 2) is present. Second, the analysis of the seven possible interaction configurations of chitosan with atomic silver was carried out under the criterion of minimum energy, as indicated in the methodological section; in addition, a cluster of silver interacting with the chitosan monomer and the effect on the systems of the acid medium (acetic acid, pH = 4.8) was analyzed.

Figure 2a,b show the optimized geometries of the chitosan monomer (MCh) and its dimer (DCh). For both structures, it was found that the minimum energy is for the neutral charge (Q = 0) and singlet multiplicity (M = 1); see Table A1. Only an increase of 0.132, 0.07, 0.14, 0.10, and 0.21 percentages of the C–C, C–N, C–O, N–H, and O–H bonds were observed in the analysis of the structural properties. The bond angles showed a significant change of approximately 1–2 degrees on average for the C–C–O, C–O–C, C–O–H, H–N–H, and N–C–H angles (Table 1). On the other hand, the numerical value of the polarity of both structures indicates their highly polar character—for DCh with a value of 6.98 D and higher than the MCh (3.59 D). This is due to the presence of a second amino group in the dimer.

One quantum parameter that indicates qualitative electronic behavior is the energy gap (ΔΕ_gap_); for both, the insulator character is presented with a value of 7.0 eV. At the same time, a high chemical reactivity value is because the chemical potential for both DCh and MCh is less than −5.0 eV (Table 1).

**Figure 3:**
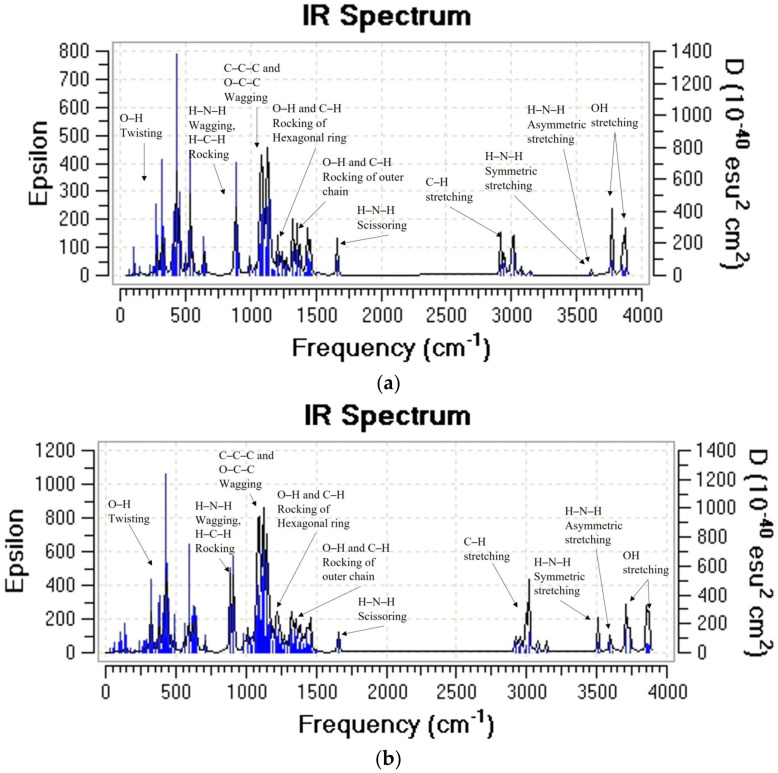
Theoretical infrared spectra of (**a**) chitosan monomer and (**b**) chitosan dimer.

Both structures present high stability (cohesion energy: MCh= −5.4 eV/atom and DCh = −5.5 eV/atom), the dimer being more stable by 0.1 eV. These results are close to those reported by Upma et al. [38], who studied the effect of cohesion energy on the position of the amino functional group in the DCh. However, this study showed that at this level of theory, the MCh also presents good structural stability. In the same context, the vibrational analysis of both chemical species indicates that they are stable, since no complex frequency is observed. Figure 3 shows the theoretical infrared spectra, where the peaks are located in the following ranges: 3680–3711 cm^−1^, 3620–3590 cm^−1^, 3520–3500 cm^−1^, 3140–2900 cm^−1^, 1665–1650 cm^−1^, 1460–1400 cm^−1^, and 1390–1200 cm^−1^ are associated with the stretching of the O–H, asymmetric stretching of the H–N–H, and symmetric stretching of the H–N–H, the stretching of C–H, H–N–H scissoring, the O–H and C–H outer chain rocking link, and O–H and C–H internal chain rocking-type vibrational modes, respectively [39,40].

The peak located from 1150 to 1000 cm^−1^ shows a wagging movement of the C–C–C and for the O–C–C link of the hexagonal ring; on the other hand, the wagging mode of the H–N–H and rocking motions of the H–C–H appear at 884 cm^−1^ for the MCh (Figure 3a) and 913 cm^−1^ (Figure 3b) for the DCh. Finally, a small peak in the 590–300 cm^−1^ range, distinctive of the O–H group and the twisting type, can be seen. The work function value in both structures is high, 3.50 and 3.59 eV, for the MCh and DCh, respectively, so it is unlikely that a photocurrent will occur.

### 3.1. Structural and Electronic Properties of Chitosan–Silver Complex

**Figure 4:**
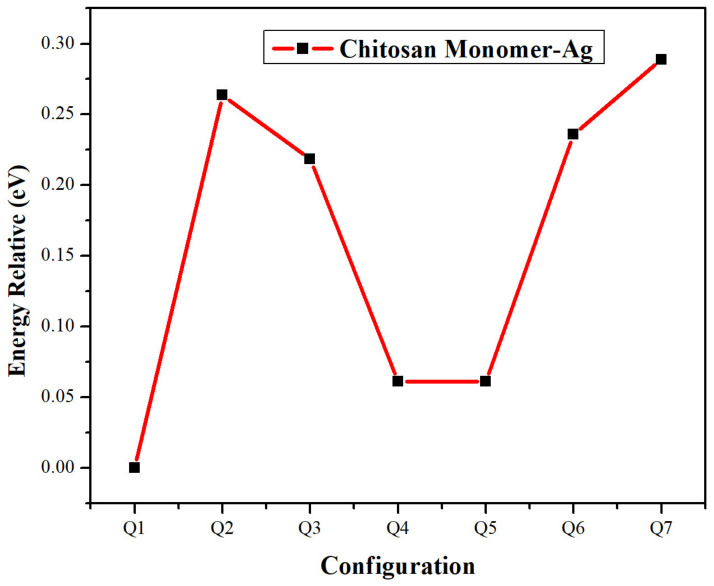
Geometrical configuration vs. relative energy of chitosan monomer and silver atom interaction.

Figure 4 presents the geometric configuration vs. relative energy concerning more negative energy configuration; we can observe the minimal energy state. Therefore, the more stable structure is Q1, because it has 0.061 eV less than the Q4 and Q5 structures, where the Ag atom interacts with the amine group from chitosan. These results agree with reports in the literature where the interaction of metallic atoms such as Cu and Na are carried out by said amino functional group, as reported [41,42].

**Figure 5:**
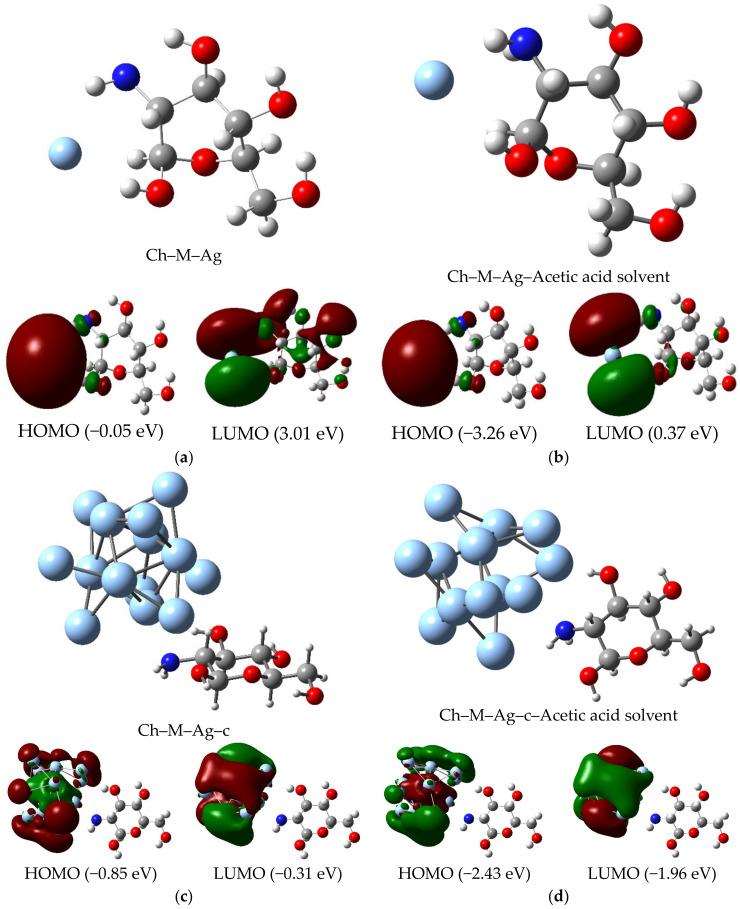
Geometrical structures of: (**a**) Ag chitosan monomer, (**b**) Ag atom–chitosan with solvent, (**c**) Ag unit cell–chitosan and (**d**) unit cell–chitosan with solvent.

**Figure 6:**
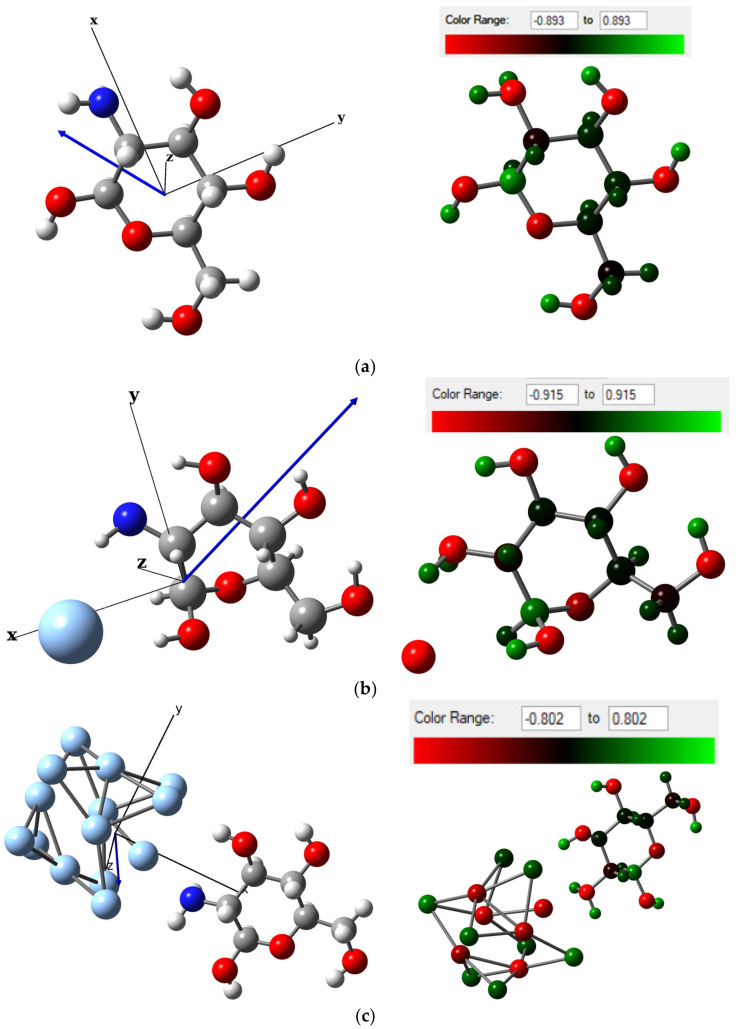
Dipole moment vector and calculated from charge NBO analysis showing charge transferring around the gas-phase systems of (**a**) Chitosan monomer, (**b**) Chitosan monomer–single silver atom complex, and (**c**) Chitosan monomer–cluster silver complex.

The re-optimized structures under the def2-TZVPP50 basis set of MCh–Ag and MCh–Ag–c are shown in Figure 5; both systems find the energy minimum on the anionic charge potential energy surface (Q = −1|e|) and multiplicity 1 and 2, respectively (Table A2), with an adsorption energy of −1.63 eV for MCh–Ag and −2.45 eV for MCh–Ag–c, indicating that the Ag chemically adsorbs the MCh in both systems (Table 1). The structural parameters, such as bond length and bond angle of the MCh–Ag and MCh–Ag–c complexes are also shown in Table 1, and only a slight change of 1.0% in the bond length of the N–H and O–H in the case of MCh–Ag concerning MCh–Ag–c. On the other hand, the dipole moment compared to the chitosan monomer (3.45 D) increases for MCh–Ag (7.1 D). It decreases for the MCh–Ag–c system (2.17 D), maintaining the covalent character, which implies that there is a charge transfer from silver to the chitosan monomer (Table 1) and through the Natural Bond Orbital (NBO) analysis images of Figure 6, said charge transfer is approximately 0.071|e|, concentrating on the hexagonal ring of the MCh. However, according to the chemical stability determined by the cohesion energy, it can be observed that the two systems are less stable than the isolated species, i.e., the MCh; however, both structures are highly stable, because the numerical values for MCh–Ag and MCh–Ag–c are −5.26 and −4.101 eV/atom, respectively (Table 1).

**Figure 7:**
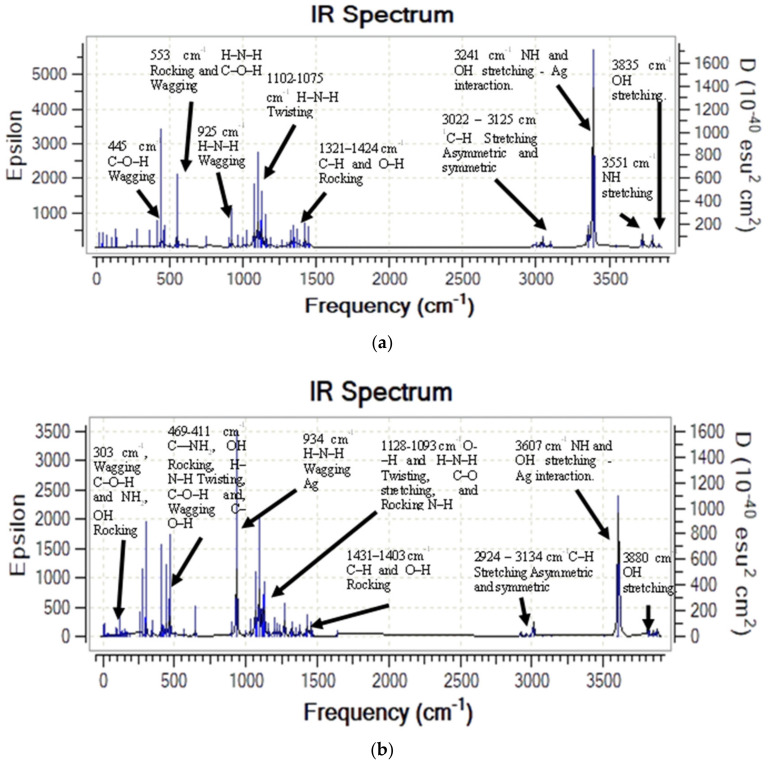
Theoretical infrared spectra of gas-phase systems: (**a**) MCh–Ag complex and (**b**) MCh–Ag–c complex.

The infrared spectra calculated from MCh–Ag and MCh–Ag–c complex vibrational analysis are shown in Figure 7. We cannot observe any complex frequency indicating that both systems are stable in the gas phase. Figure 7a shows the IR spectrum of the MCh–Ag system where the O–H, N–H, and C–H stress-type modes are located at 3835 cm^−1^, 3551 cm^−1^, and 3022–3125 cm^−1^, respectively.

However, when the MCh interacts with the Ag atoms (MCh–Ag–c) cluster, the O–H tension shifts to higher wave numbers of 45 cm^−1^. Although the C–H tension undergoes a shift to lower wave numbers (Figure 7b), both systems present a high-intensity peak located at 3241 cm^−1^ for the MCh–Ag and 3607 cm^−1^ MCh–Ag–c due to the interaction of the O–H functional group of the MCh hexagonal ring and N–H with the silver; these results confirm the experimental studies by An et al. [43] where it is mentioned that both the amino group of MCh and OH functional group interact with the silver atom.

On the other hand, the deformation rocking vibration modes of the O–H and the C–H of the MCh–Ag are located in the ranges 1321–1424 cm^−1^ and 1431–1403 cm^−1^ of the MCh–Ag and MCh–Ag–c complex, respectively, while the twisting vibrational mode due to the H–N–H, O–C–H are between 1128 and 1075 cm^−1^, and the intense peaks are due to the interaction with Ag and MCh with the respective wagging movement of the H–N–H bond located at 925 and 934 cm^−1^, for the MCh–Ag and 3607 cm^−1^ and MCh–Ag–c, respectively. A small peak in the 469–411 cm^−1^ range is due to a combined rocking movement of the C–H–N–H and O–H, twisting of the OH–C, and wagging C–O–H to MCh–Ag–c, while MCh–Ag is located at 445 cm^−1^.

**Figure 8:**
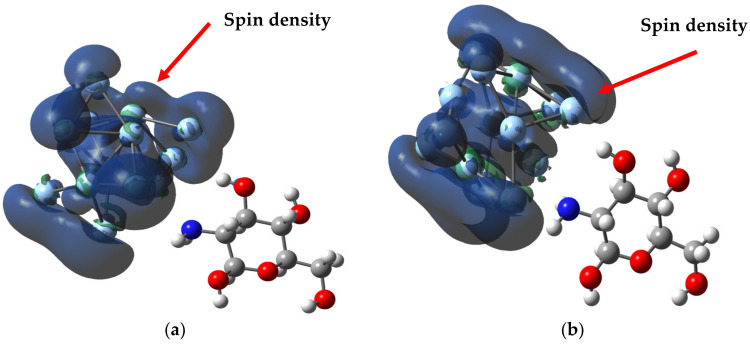
Spin density surface (0.004) for acetic acid systems: (**a**) MCh–Ag–cluster complex and (**b**) The MCh–Ag–c–Solv.

Regarding the electronic properties of the MCh–Ag and MCh–Ag–c complex shown in Table 1, it can be seen that chitosan undergoes a transition in its electronic behavior from an insulator to a semiconductor-like character when it interacts with the Ag due to the ΔΕ_gap_ value. For the MCh–Ag, it is 3.1 eV, and for the MCh–Ag–c, it is 0.55 eV. In comparison, the MCh has a gap of 7.18 eV; this can be explained by Figure 5a,b, where the density of the HOMO is Ryd(3p) type for MCh–Ag (more significant contribution of s-type orbitals of Ag and p-character orbitals of N and O) and Ryd (5d) type for MCh–Ag–c (more outstanding contribution of d-type orbitals from Ag). At the same time, the LUMO is of the Cor(1S) MCh–Ag type (contribution of p-type orbitals from both silver and O and N) and other Cor(3d) type (contribution of d-type orbitals of both silver and O and N) and are concentrated on the silver cluster, which explains the reduction of said value of the energy gap [44]. On the other hand, since the MCh–Ag–c system was stabilized in anionic charge (Q = −1|e|) and multiplicity 2, this system presents magnetic properties, with a magnetic moment of 1.0 µB, where the silver atoms are primarily responsible for magnetism. This can be confirmed by the spin density surface images shown in Figure 8a.

**Figure 9:**
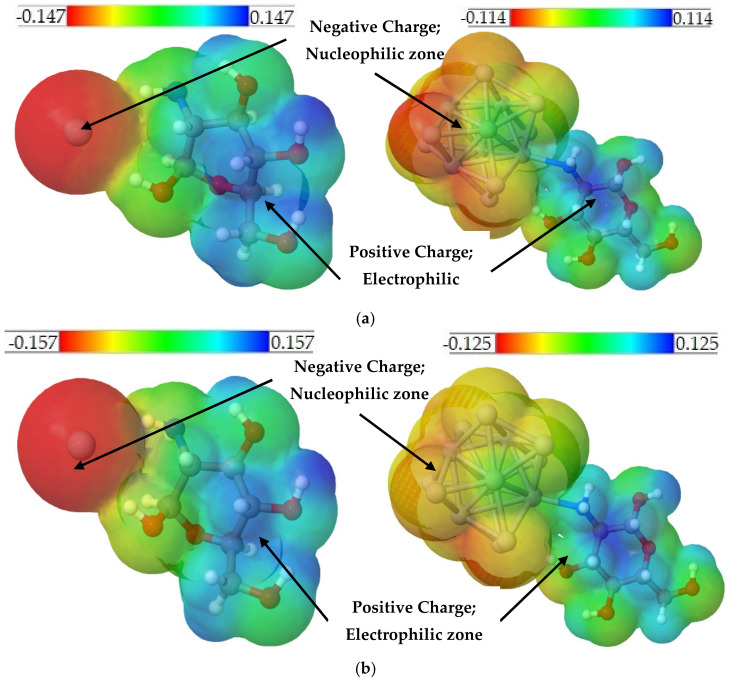
MEP iso-surfaces where the charge localization over the systems can be observed: (**a**) Gas phase and (**b**) Acetic acid.

The chemical reactivity determined by the value of the chemical potential of the MCh–Ag is 2.21 eV, while for the MCh–Ag–c it is −1.16 eV. It increases, compared to the MCh. These results support those already reported in the literature, where it is mentioned that the chitosan biopolymer involves silver nanoparticles [45,46]. In the case of the work function, its value is reduced to 0.05 eV for the MCh–Ag system and 0.85 eV for the MCh–Ag–c system (Table 1) compared to the chitosan monomer, indicating that these systems are suitable for the development of light-emitting diode systems. Additionally, the MEP iso-surface obtained by the Jmol program [47] (Figure 9a) of the structures shows us that the zone of most remarkable reactivity with a concentration of electric charge is in the silver atoms, which indicates that this zone is nucleophilic in both systems.

### 3.2. Effects of Solvent (Acetic Acid) on Chitosan–Silver Complex

The known effect of the acid medium on the complex formed by the silver cluster and the MCh in situ synthesis methods of silver nanostructures when the acid dissolves chitosan to conform biopolymer films with silver nanostructures is being developed experimentally [48].

A significant structural effect can be observed due to acetic acid on the MCh–Ag and MCh–Ag–c complexes, as shown in Figure 5b,d, i.e., it can be seen in Table 1 that this acid medium causes the attractive energy of both atomic silver and the silver cluster on the MCh to increase by 54% (for the MCh–Ag, the Ead = −1.63 eV) and 30% for MCh–Ag–c with Ead = −3.53 eV. This is because acetic acid gives chitosan the chelating character to be able to wrap both atomic silver and the cluster to stabilize the silver [49].

**Table 1:** Bond length (BL; Å), bond angle (BA; °), Energy gap (ΔΕ_gap_; eV), Dipolar moment (DM; Debye), Chemical potential (µ; eV), Cohesion energy (CE; eV/atom), Work function (WF; eV) Affinity electronic (AE; eV), Ionization potential (IP; eV) and Adsorption energy (AdE; eV) for Chitosan monomer, chitosan dimer, chitosan monomer–Ag and chitosan monomer–Ag clusters.

Structures	BL	BA	ΔΕ_gap_	DM	μ	CE	AE	IP	WF	AdE
Chitosan	-	-				−5.1	-			-
Ag–c	2.79 (Ag-Ag) 2.53 [29,50]	-	1.3 2.2 [30] 0.02 [29]	-	−7.58	−1.53 1.8 [30]	-		4.43 4.26–4.73 [51]	-
MCh monomer	1.519 (C–C), 1.10 (C–H), 1.453 (C–N), 1.408 (C–O), 1.014 (N–H), 0.962 (O–H)	C–C–C (110.83), C–C–H (108.46), C–C–N (112.39), C–C–O (109.86), C–N–H (110.3), C–O–C (113.38), C–O–H (106.29), H–C–H (108.31), H–N–H (106.99), N–C–H (108.01), O–C–H (109.95), O–C–O (108.79)	7.21	3.45	−6.69	−5.40	−2.32	−9.52	6.95	-
MCh Dimer	1.52 (C–C), 1.10 (C–H), 1.452 (C–N) 1.41 (C–O), 1.015 (N–H), 0.964 (O–H)	C–C–C (110.83), C–C–H (108.56), C–C–N (112.32), C–C–O (109.80), C–N–H (110.19), C–O–C (115.07), C–O–H (105.86), H–C–H (108.26), H–N–H (107.87), N–C–H (107.42), O–C–H (109.38), O–C–O (108.63)	7.01	6.98	−5.93	−5.5	−2.42	−9.43	3.50	-
MCh–Ag	1.53 (C–C), 1.10 (C–H), 1.45 (C–O), 1.47 (N–C), 1.03 (N–H), 1.01 (0–H)	C–C–C (80.89), C–C–H (108.56), C–C–N (114.41), C–C–O (99.41), C–N–H (110.19), C–O–C (114.80), C–O–H (83.73), H–C–H (108.64), H–N–H (109.97), N–C–H (104.95), O–C–H (109.66), O–C–O (107.84)	3.1	7.1	2.96	−5.26	3.01	−0.05	0.05	−1.63
MCh–Ag–Cluster	1.52 (C–C), 1.10 (C–H), 1.45 (C–O), 1.47 (N–C), 1.02 (N–H), 1.00 (O–H), 2.43 (Ag–N)	C–C–C (110.83), C–C–H (108.46), C–C–N (112.39), C–C–O (109.86), C–N–H (110.3), C–O–C (113.38), C–O–H (106.29), H–C–H (108.31), H–N–H (106.99), N–C–H (108.01), O–C–H (109.95), O–C–O (108.79)	0.55	2.17	−1.16	−4.10	−0.31	−0.85	0.85	−2.45
MCh–Ag-Acetic acid solvent	1.53 (C–C), 1.10 (C–H), 1.45 (C–O), 1.48 (N–C), 1.03 (N–H), 1.00 (0–H)	C–C–C (110.83), C–C–H (108.46), C–C–N (112.39), C–C–O (109.86), C–N–H (110.3), C–O–C (113.38), C–O–H (106.29), H–C–H (108.31), H–N–H (106.99), N–C–H (108.01), O–C–H (109.95), O–C–O (108.79)	3.64	9.96	−2.89	−5.32	0.37	−3.26	3.26	−3.56
MCh–Ag–c-Acetic acid solvent	1.53 (C–C), 1.10 (C–H), 1.46 (C–O), 1.46 (N–C), 1.02 (N–H), 1.01 (O–H)	C–C–C (110.83), C–C–H (108.46), C–C–N (112.39), C–C–O (109.86), C–N–H (110.3), C–O–C (113.38), C–O–H (106.29), H–C–H (108.31), H–N–H (106.99), N–C–H (108.01), O–C–H (109.95), O–C–O (108.79)	0.48	2.97	−4.39	−4.11	−1.95	−2.43	2.44	−3.53

**Figure 10:**
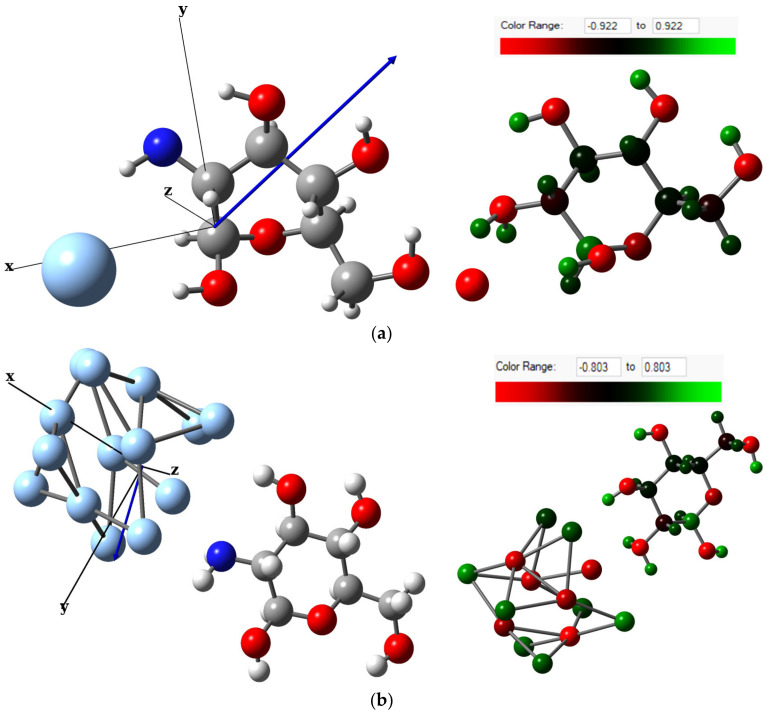
Dipole moment vector and calculated from charge NBO analysis showing charge transferring around the acetic acid systems of (**a**) Chitosan monomer–single silver atom complex with solvent and (**b**) Chitosan monomer–cluster silver complex with solvent.

The MCh–Ag–c–Solv system, as in the case of the gas phase, presents magnetic properties with a magnetic moment of 1.0 µB, where the spin density is located mainly in the silver atoms and partially in the O atoms of the monomer (Figure 8b). The monomer structure was somewhat altered, presenting a reduction of 0.65, 1.55, and 3.8% in the C–C, N–H, and OH bond lengths, respectively, due to the 3% increase for the C–O bond lengths, compared to the MCh (the bond angles do not present significant alteration, Table 1). The dipole moments for the systems increase by 9.96 D for the MCh–Ag–Solv system, and 2.97 for MCh–Ag–c–Solv compared to the gas-phase system, and, due to the charge transfer of 0.08|e| silver, is given in the direction of the atom towards chitosan, concentrating on the amino group (Figure 10).

**Figure 11:**
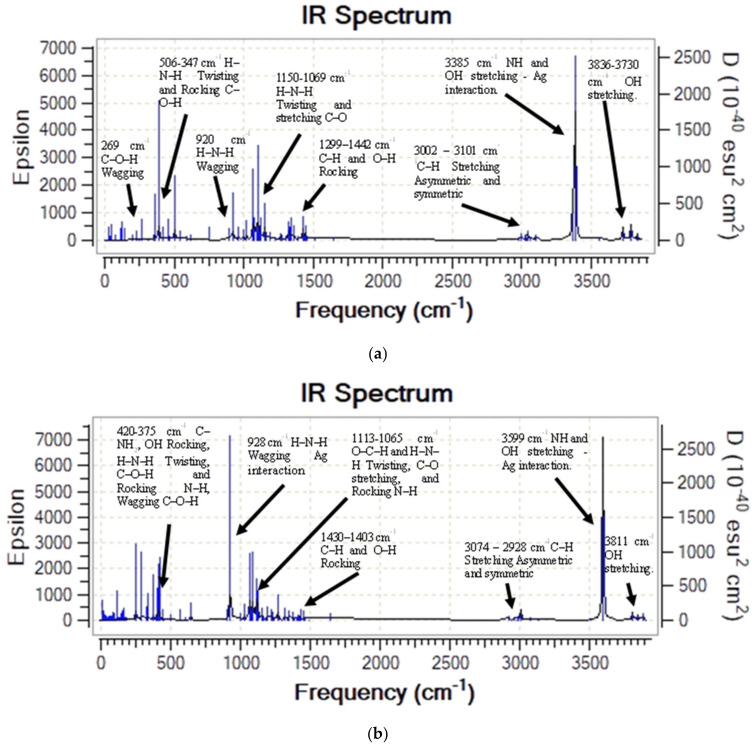
Theoretical infrared spectra of acetic acid systems: (**a**) MCh–Ag atom complex and (**b**) MCh–Ag–cluster complex with solvent.

Vibrational analysis shows that both systems are stable as they do not present negative vibration frequencies. Figure 11a shows the IR spectrum of the MCh–Ag–Solv system, which shows that the tension vibration modes of O–H, N–H, and C–H are in the ranges of 3836–3730 cm^−1^, 3385 cm^−1^, and 3002–3101 cm^−1^, respectively. However, when the MCh interacts with an Ag cluster (MCh–Ag–c–Solv), the O–H and H–N–H tensions shift to higher wavenumbers; i.e., they are located at 3811 cm^−1^ and 3599 cm^−1^, while the C–H tension undergoes a shift to lower wavenumbers in both systems (Figure 11a,b). The IR spectra show peaks as more intense at 3385 cm^−1^ and 3599 cm^−1^ of symmetric and asymmetric tension for NH and OH interactions with a silver atom. This is due to the acetic acid solvent. The deformation vibrational modes for the MCh–Ag–Solv system are found in the regions 1299–1441, 1150–1069, 920, 506–347, and 269 cm^−1^ associated with C–H and O–H rocking, H–N–H twisting, H–N–H wagging, a combination of H–N–H–C twisting and C–O–H rocking, and C–O–H wagging, respectively (Figure 11a). In the same sense, the deformation modes of the MCh–Ag–c–Solv system (Figure 11b) are located at 1430–1403 cm^−1^ for C–H and O–H rocking, 1113–1065 cm^−1^ of O–C–H and H–N–H twisting, and N–H rocking, 928 cm^−1^ for H–N–H wagging interaction with a silver atom, and finally in the interval 420–375 cm^−1^ due to a combination between C–NH_2_, OH rocking, H–N–H twisting, N–H rocking, and the C–O–H wagging functional group.

Regarding the electronic properties, the MCh–Ag–Solv and MCh–Ag–c–Solv systems present a semiconductor character because the ΔΕ_gap_ value is 3.64 eV and 0.48 eV, respectively; this is because, like the systems in the gas phase, the densities of the HOMO and LUMO orbitals are distributed over the silver atoms (see Figure 5b,d). The chemical reactivity given by the value of the chemical potential indicates that acetic acid increases the chemical reactivity by 3% for MCh–Ag–Solv and 70% for MCh–Ag–c–Solv, indicating that this acid medium promotes an increase in charge transfer in the systems (Table 1). The cohesion energy of the systems both in the gas phase and in acetic acid indicates that stability tends to increase with the addition of a metallic silver cluster and with the solvent (Table 1). Finally, the work function is altered for the MCh–Ag–c–Solv with an increase of 1.59 eV and 3.21 eV to MCh–Ag–Solv compared to the gas-phase systems. Still, it maintains an excellent optical activity to the flow of electrons. The iso-surface MEPs, like the gas-phase systems (Figure 9b), show us that the nucleophilic area is in the silver atoms.

## 4. Conclusions

DFT calculations were used to study the structural, electronic, and optical properties of the MCh–Ag and MCh–Ag–c systems with the chemical composition C_6_O_5_H_13_N–Ag and C_6_O_5_H_13_N–Ag_14_, respectively, and the effect of acetic acid as a solvent medium. The structural stability of the systems is high and increases with the impact of acetic acid. Our results agree that chitosan has already been experimentally reported as a stabilizing agent for silver nanostructures. According to their quantum descriptors, it was found through the analysis of the global chemical potential that the systems have moderate chemical reactivity and high polarity, so these systems can be considered feasible for the design of light-emitting diodes. In the same way, the design of optoelectronic devices using these systems can occur because they present a moderate value of work function. On the other hand, the doublet state of the C_6_O_5_H_13_N–Ag_14_ complex in the gas phase and with a solvent such as acetic acid suggests that the system may have potential use for removing metal ions with ferromagnetic properties. The systems can even be used as nucleophilic agents since the MEP iso-surfaces show that the negative charge is concentrated in the silver atom.

## Data Availability

The data supporting reported results are available from the corresponding author, A.R.J., upon reasonable request.

## Appendix A

**Table A1:** Analysis of the global charge (Q) and multiplicity (M = 2ST + 1) for Chitosan monomer and dimer at HSEh1PBE/6-311G(d,p) level DFT theory.

System	Charge	Multiplicity	Energy (a.u.)
MCh monomer	Q = 0	M = 1	−666.82740955
		M = 3	−666.66173446
		M = 5	Not converged
	Q = 1	M = 2	−666.52571752
		M = 4	−666.60926898
		M = 6	−666.21251597
	Q = −1	M = 2	−666.76215072
		M = 4	−666.35597400
		M = 6	−666.42863642
MCh dimer	Q = 0	M = 1	−1257.28370138
		M = 3	−1257.10948524
		M = 5	−1256.94272493
	Q = 1	M = 2	−1256.99318357
		M = 4	−1256.82376338
		M = 6	−1256.67429205
	Q = −1	M = 2	−1257.22834634
		M = 4	Not converged
		M = 6	−1257.06361985

**Table A2:** Q values for the different systems at HSEh1PBE/DGDZVP method.

System	Charge	Multiplicity	Energy-Sn Solvent (a.u)
MCh–Ag	1	1	−5838.64279216
		3	−5838.50268676
		5	−5838.38555019
	0	2	−5838.78969058
		4	−5838.61281027
		6	−5838.46723759
	−1	1	−5838.80708882
		3	−5838.76019335
		5	−5838.65024934
MCh–Ag–cluster	1	2	−73,125.31239650
		4	−73,125.18277190
		6	−73,125.11091140
	0	1	−73,125.31239650
		3	−73,125.29911230
		5	−73,125.32097840
	−1	2	−73,125.3655746
		4	−73,125.3523194
		6	−73,125.3547286
